# Comparison of neonatal outcomes following progesterone use during ovarian stimulation with frozen-thawed embryo transfer

**DOI:** 10.1038/s41598-017-08472-2

**Published:** 2017-08-10

**Authors:** Xiuxian Zhu, Hongjuan Ye, Yonglun Fu

**Affiliations:** Department of Assisted Reproduction, Shanghai Ninth People’s Hospital, Shanghai Jiaotong University School of Medicine, 639 Zhizaoju Rd., Shanghai, 200000 China

## Abstract

Progesterone soft capsules (brand name: Utrogestan) were demonstrated to be an effective oral alternative to prevent premature LH surges both in normal-ovulatory and polycystic ovarian syndrome (PCOS) patients. However, its safety in terms of neonatal outcomes is unclear. To evaluate whether Utrogestan use increase the risk of adverse neonatal outcomes compared with short protocol in patients undergoing IVF/ICSI treatments in combination with frozen-thawed embryo transfer (FET), we performed a retrospective analysis including 1008 FET cycles, with embryos originated from either Utrogestan + hMG protocol (n = 499), or short protocol (n = 509), which led to 546 live-born infants. The neonatal characteristics regarding preterm birth (PTB), low birth weight (LBW), gestational age and mode of delivery were comparable in the two groups. The incidence of live-birth defect was 0.68% (2/293) in the Utrogestan + hMG protocol compared with 0.79% (2/253) in the short protocol. No early neonatal death or intrauterine death were recorded in either group. To date, the data do not indicate an elevated rate of abnormality at birth after progesterone use during ovarian stimulation but further study with larger populations is needed to confirm these results.

## Introduction

During the past 40 years, large scale number of infertile couples have babies through assisted reproductive technology (ART),inconsist of *in vitro* fertilization (IVF), intracytoplasmic sperm injection (ICSI), *in vitro* maturation (IVM), embryo cryopreservation, frozen thawed ET, and preimplantation genetic diagnosis (PGD). ART pregnancies approximately accounts for 1.7–4% of births in developed countries, in contrast with 1.013% of births according to a report from a 2011 survey on the proportion of births born after ART in mainland China^1^. With the rapid development of technology and service, the proportion of ART pregnancies is steadily rising every year. As a result, the safety of ART procedures has attracted more and more attention due to the possible impact on pregnancy complications and live-birth outcomes in infants conceived by ART.

The introduction of controlled ovarian hyperstimulation (COH) has been a vital clinical milestone to increasing the success rates of ART by virtue of increasing the number of oocytes retrieved. However, premature luteinizing hormone (LH) surge was a major issue during COH, which may result in cycle cancellation owing to premature luteinization and ovulation. The usage of gonadotropin- releasing hormone agonist (GnRH-a) and GnRH antagonist has sharply reduced the occurrence of premature LH surges. More importantly, these regimens has been accepted as being safe because the incidence of obstetrical complications and congenital malformations were similar to those conceived spontaneously based on a series of follow-up studies^2^.

However, various disadvantages still exist, e.g., the complexity of achieving consistent downregulation, an increased risk of ovarian hyperstimulation syndrome (OHSS) from a human chorionic gonadotrophin (hCG) trigger, and expensive cost, suboptimal oocyte or embryo quality induced by premature luteinization^3–5^, which inspired us to explore convenient alternatives to prevent premature LH surges. When fresh embryo transfer (ET) was the norm in IVF, steroidal preparation could not be considered for use during COH because of its negative impact on endometrial receptivity though it was powerful modulators of pituitary gonadotropin (Gn) secretion^6, 7^. Since advanced vitrification techniques made superior quality cryopreserved embryo and precise thawing possible, frozen-thawed embryo transfer (FET) has been widely used in recent years. What’ more, the “freeze-all” strategy has been recommended due to the improved pregnancy and delivery outcomes^8–10^.

In combination with the “freeze-all” strategy, we corroborated luteal phase ovarian stimulation (LPS)—initiating ovarian stimulation in the luteal phase using letrozole and Gn—was able to achieve consistent LH suppression, thus, we postulated high doses of progesterone may transiently be feasible to suppress pituitary gland secretions^11^. Moreover, our large, retrospective study of 2,060 live-born infants found that the incidence of live-birth defects in patients undergoing COH with LPS (1.02%) was comparable with the short protocol (0.64%)^12^.

A hypothesis was proposed that progesterone delivered from the early follicular phase may be used to suppress premature LH surges. Soon afterwards, we verified progesterone soft capsule (brand name: Utrogestan), as a kind of natural micronized progesterone which was capable of detecting in serum after being taken orally or vaginally was an effective oral alternative for inhibiting premature LH surges both in normal-ovulatory and polycystic ovarian syndrome (PCOS) patients^13–16^. Furthermore, compared with the short protocol, no statistically significant differences in the pregnancy outcomes of FET were found in normal-ovulatory patients while biochemical pregnancy rate, clinical pregnancy rate, and implantation rate increased in PCOS patients undergoing COH with Utrogestan + hMG protocol^13, 14^. Though more studies were needed to evaluate the usage of Utrogestan from the early follicular phase, it is a new choice for patients undergoing IVF/ICSI treatments following embryo cryopreservation.

To date, hundreds of infants were born by virtue of this novel regimen. Accompanying the desire to continuously optimize this novel protocol is a concern about whether it has any adverse effect on the safety of babies and mothers. However, there are no relevant studies addressing this problem. The aim of this study was to assess neonatal outcomes after COH using Utrogestan + hMG protocol in comparison with short protocol in women undergoing IVF/ICSI treatments following a “freeze-all” strategy.

## Results

**Figure 1:**
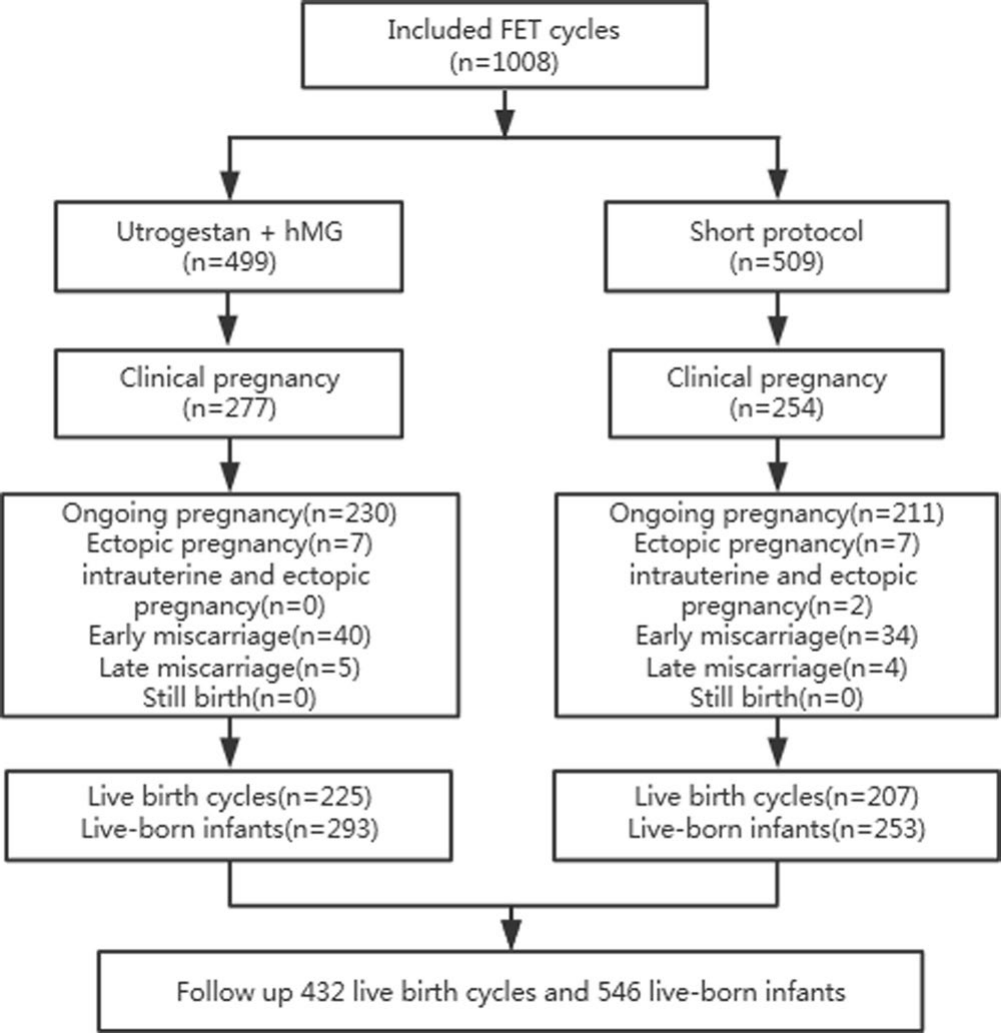
Flowchart of the study.

The sample of 531 pregnancy cycles resulting from 1008 FET procedures, from January 1, 2014 to December 31, 2014, consisted of 441 ongoing pregnancies, 17 ectopic pregnancies, 2 heterotrophhic pregnancies, and 74 early miscarriages, resulting in a total of 546 live-born infants (432 live-birth cycles). Among them, 277 pregnancies originating from 499 FETs led to the birth of 293 live-born neonates after treatment with Utrogestan + hMG protocol group; 254 pregnancies originating from 509 FETs led to the birth of 253 live-born babies after treatment with the short protocol group (details of group distribution profiles are presented in the flow chart in Fig. 1).

The characteristics of women included, such as maternal age, body mass index, infertility duration, infertility diagnosis, proportion of nullipara, endometrial preparation, and type of embryos transferred were comparable in the two groups (Table 1).

**Table 1:** Baseline characteristics of sample population stratified by ovarian stimulation method.

Outcome	Study group (Utrogestan + hMG)	Control group (Short protocol)	P
Total FETs (n)	499	509	
Patients (n)	376	381	
Nullipara	339(90.16%)	349(91.6%)	0.491
Maternal age (y)	31.87 ± 4.08	32.11 ± 3.62	0.322
Body mass index (kg/m^2^)	21.81 ± 3.24	21.6 ± 2.94	0.189
Infertility duration (y)	3.33 ± 2.15	3.93 ± 2.95	0.346
Infertility diagnosis			0.104
Tubal factor	212	230	
Male factor	79	90	
Combination of factors	48	39	
Unknown factor	37	22	
Endometrial preparation			0.299
Natural cycle	222	233	
Mild stimulation	245	232	
Hormone therapy	32	44	
Thawed embryos (n)	985	981	
Viable embryos after thawed (n)	972	972	
Type of embryos			0.183
Cleavage-stage ETs	891	873	
Blastocyst ETs	81	99	

Although the biochemical pregnancy rate (60.32% vs. 54.81%) and clinical pregnancy rate (55.51% vs. 49.9%) in the Utrogestan + hMG protocol group was higher than those in the short protocol group, these differences were not statistically significant (P > .05).The implantation rate (37.14% vs.34.47%), early miscarriage rate (14.44% vs. 13.39%), late miscarriage rate (1.81% vs. 1.57%), multiple pregnancy rate (32.85% vs. 31.1%), ectopic pregnancy rate (2.53% vs. 2.76%), intrauterine and ectopic pregnancy rate (0 vs. 0.79%), ongoing pregnancy rate (46.09% vs. 41.45%) were comparable in the two groups (Table 2).

**Table 2:** Pregnancy and neonatal outcomes stratified by ovarian stimulation method.

Outcomes	Study group (Utrogestan + hMG)	Control group (Short protocol)	P
**Pregnancy outcomes of FET**			
Biochemical pregnancy rate	60.32%(301/499)	54.81%(279/509)	0.077
Clinical pregnancy rate per transfer	55.51%(277/499)	49.9%(254/509)	0.075
Implantation rate	37.14%(361/972)	34.47%(335/972)	0.219
Early miscarriage rate	14.44%(40/277)	13.39%(34/254)	0.726
Late miscarriage rate	1.81%(5/277)	1.57%(4/254)	0.837
Multiple pregnancy rate	32.85%(91/277)	31.1%(79/254)	0.666
Ectopic pregnancy rate	2.53%(7/277)	2.76%(7/254)	0.869
Intrauterine and ectopic pregnancy rate	0%(0/277)	0.79%(2/254)	0.139
Ongoing pregnant rate per transfer	46.09%(230/499)	41.45%(211/509)	0.138
Preterm birth rate	17.33%(39/225)	12.08%(25/207)	0.124
Live-birth rate per transfer	45.09% (225/499)	40.67%(207/509)	0.156
**Neonatal outcomes**			
Gestational age (wk)			0.265
<28	1	0	
28 ≤ age < 37	38	25	
≥37	185	182	
≥42	1	0	
Mode of delivery			0.891
Vaginal	60	54	
Caesarean	165	153	
Live born infants (n)	293	253	0.071
Single newborns (n)	152	153	
Single birth weight (g)	3327.82 ± 481.4	3282.79 ± 520.53	0.559
Single birth length (cm)	49.92 ± 1.49	48.9 ± 6.94	0.902
Twin newborns (n)	146	108	
Twin birth weight (g)	2591.32 ± 565.99	2551.15 ± 463.29	0.98
Twin birth length (cm)	47.62 ± 6.2	48.04 ± 1.99	0.441
Birth weight < 2,500 g	26.96%(79/293)	22.92% (58/253)	0.278
Early neonatal death	0	0	

The live-birth rate per transfer, PTB rate, and post-term pregnancy rate in the Utrogestan + hMG protocol group (45.09%, 17.33%, and 0.44%, respectively) are not statistically different from those in the short protocol group (40.67%, 12.08%, and 0, respectively), What’s more, the mode of delivery was comparable in the two groups. Similarly, a nonsignificant difference was found among the 2 groups for the incidence of multiple delivery, despite the fact that more multiple pregnancies in the Utrogestan + hMG protocol group was deliveried than that in the short protocol group. No significant differences were found in the birth length and weight for the two groups, both in the single newborns and twins newborns. Similarly, LBW rate (22.96%vs. 22.92%) were comparable in the two groups. In addition, no early neonatal death was found in the participants (Table 2).

A total of 4 cases (0.73%) qualified as having congenital defects in all live-born infants, according to the definition in the International Classification of Diseases. Defects were observed in 2 of 293 (0.68%) in the Utrogestan + hMG protocol group, and 2 of 253 (0.79%) in the short protocol group; however, these values were all very low, and the differences was statistically significant (P > 0.05). Comparisons between groups of birth defects according to neonatal gender, singletons, and multiples were carried out and reached the undifferentiated outcomes presented in Table 3. The detailed breakdown of detected malformations according to the various organ systems is also presented in Table 3.

**Table 3:** Types of congenital malformations among 546 live-born infants.

Outcomes	Study group (Utrogestan + hMG)	Control group (Short protocol)
Number of birth defects	2	2
Number of deliveries		
Singletons	0	1
Multiples	2	1
Birth defects, by gender		
Male	1	1
Female	1	1
Malformation type		
Circulatory system	Q21.102: atrial septal defect (1)	Q21.001: ventricular septal defect (1)
Digestive system	K40.903: indirect inguinal hernia (1)	/
Motor system	/	Q69.901: polydactyly (1)

## Discussion

Our previous studies have confirmed the oral delivery of Utrogestan is an effective way to block premature LH surges with component oocytes and embryos. However, with each new regimen, it must be shown not only that it is effective but also that it does not adversely affect the children’s health. The latter is the most important as the ultimate objective with ART is to efficiently achieve healthy live birth outcomes. The results of our study do not indicate an elevated rate of abnormality at birth after Utrogestan + hMG protocol compared with short protocol in patients undergoing IVF/ICSI treatments with FET.

Since the application of ART, concerns have been raised regarding both its safety and its effect on fetal well-being. Compared with fertile women who conceived spontaneously, a significantly higher risk of adverse obstetric outcomes such as perinatal mortality, preterm delivery (PTD), low-birth-weight (LBW), very-low-birth-weight (VLBW), and small-for-gestational-age (SGA) infants was observed in pregnancies following ART^17, 18^. In addition, a recent meta-analysis including birth defect information on 92 671 ART infants confirmed an increased birth defect risk exists in ART infants^19^. Some researchers deem the risk may be attributable to patient characteristics related to infertility, including older age, higher BMI, longer infertile time, PCOS, serious endometriosis and other related etiology of infertility^20–24^, while others insist it is related to the treatment-related factors, such as embryo culture conditions, cryopreservation technique, endometrial preparation, COH protocol, and other potentially relevant parameters^25–28^. In our study, not only the patient characteristics in terms of maternal age, body mass index, infertility duration, infertility diagnosis, proportion of nullipara were comparable, but also type of embryos transferred, the method of fertilization, endometrial preparation, luteal support and other routine laboratory procedure were similar in the two groups, thus COH protocol differs.

Compared with natural conception, COH exposure is correlated with superphysiologic steroid hormone environment may result in altered endometrial development, in consist of histologic advancement, premature down-regulation of the P receptor, an abbreviated luteal phase, glandular-stromal dyssynchrony, genomic dysregulation, altered leukocyte localization and activation, premature nucleolar channel formation, advanced angiogenesis, increased blood vessel density, and reduced endometrial blood flow^26^. Hu *et al*. corroborated that the high E_2_ environment resulted from COH is relevant to the increased risks of LBW and SGA. Elevated E_2_ level has an adverse effect on endometrial receptivity and implantation process, meanwhile, it may impair spiral artery invasion in the first trimester and compromise uteroplacental blood flow at term, leading to restricted fetal growth. Therefore, embryo cryopreservation was recommended when the maternal E_2_ level is extremely high on the hCG administration day and luteal support with E_2_ supplemention should be cautious^29^. The “freeze-all” strategy was indispensable because the usage of Utrogestan from the early follicular phase will affect endometrial development.

In addition, it was substantiated the degree of histologic advancement increased when P levels are prematurely elevated^30^. There has been contradictory results in previous literatures that pertain to the effects of P on the outcomes of IVF/ICSI. The occurrence of a preovulatory increase in serum P levels was reported to 12.4–52.3% in down-regulation protocol^31^. Some studies have concluded that P elevation has an detrimental effect on the pregnancy and implantation rate in patients undergoing fresh ET following GnRH agonist and antagonist cycles^32, 33^, whereas others claimed exert no impact on pregnancy outcomes^34, 35^. A better outcome was even observed in patients with PCOS or donor oocyte^36, 37^. Adverse effects on IVF outcomes due to elevated P level may be associated with its effect on oocyte, embryo quality and/or endometrial receptivity. A recent meta-analysis including more than 60,000 cycles conducted by Venetis *et al*. confirmed the main deleterious effect of P elevation acts on endometrial receptivity^38^. In accordance with a functional genomics analysis study, P elevation was proved to impose a detrimental effect on endometrial gene expression^39, 40^. The adverse impact of the high progesterone level on the endometrium can be neglected as all patients underwent FET in our study.

It was known that early embryonic survival, the establishment and maintenance of pregnancy, and even growth abnormalities is closely related to oocyte quality^41^. Thus, whether the administration of Utrogestan from the early follicular phase had detrimental effect on oocyte or embryo quality was a major concern. The effects of progesterone on oocyte maturation and embryo development was unclear both *in vitro* and *in vivo*. In the investigation performed by Salehnia *et al*., where different progesterone concentrations (10, 38, 50, 100 μM) was added to the *in vitro* oocyte maturation (IVM) media of mouse GV oocytes, the maturation rate decreased in a dose dependent manner and the GV arrested rates increased^42^. Similar reduction of oocyte maturation rate was observed in the experiment made by Fukui *et al*. when supplemented progesterone to IVM culture systems of bovine^43^. Silva *et al*. found that the rate of blastocyst formation was sharply reduced by the exposure of bovine cumulus oocyte complexes to progesterone^44^. In contrast, no significant differences was found in canine oocyte maturation among the four groups with distinct hormonal environment^45^. Carter *et al*. have shown that addition of progesterone to culture medium did not affect the proportion of *in vitro* matured/*in vitro* fertilized zygotes that developed to the blastocyst stage *in vitro* ^46^. In addition, it is intriguing that better results was obtained with the exposure of progesterone. Supplementation of canine oocyte culture media with progesterone and E2 was found to stimulate oocyte maturation^47, 48^. Zhang and Armstrong reported that the addition of progesterone to porcine oocyte maturation medium could improve both fertilization and cleavage rates^49^. Furthermore, it was validated the high progesterone concentration in follicular fluid were associated with better embryo development in humans and rhesus monkeys^50^. Our previous research confirmed this possibility to some extent, as the viable embryo rate per oocyte retrieved was significantly improved both in nomal-ovulatory patients and PCOS patients using Utrogestan + hMG protocol.

The serum P values increased swiftly after Utrogestan administered orally, with a range of 0.9–47.8 ng/mL reported in our previous studies, and then maintained at stable concentrations following continuous delivery. The absorption and elimination of Utrogestan were dose independent, thus a considerable inter-individual variation was recognized^16^. Though it was shown that oocytes aspirated during the luteal phase, a physiological context of high progesterone with an average peak level of 11.1 ng/mL, are capable of maturing *in vitro* and being fertilized^11^, no powerful evidence has proven the safety of high progesterone exposure in the early follicular phase for offspring that originate from this ovarian-stimulation regimen. Therefore, we followed up on the live-birth defects of infants born from Utrogestan + hMG protocol. In our study, the clinical pregnancy rate, implantation rate, ongoing pregnancy rate, live birth rate were comparable in the two groups, which illustrated that the high context of progesterone did not impair the pregnancy outcomes. Furthermore, various birth characteristics in consist of weeks of gestation, PTB rate, postterm delivery rate, birth weight and length, odds of LBW, multiple delivery rate, early neonatal mortality, and incidence of live-birth defects, were similar in the two groups. The results of this study indicated that the artificial high context of progesterone by taking Utrogestan was not associated with any significant increase in risk of pregnancy or neonatal outcomes compared with short protocol in patients undergoing IVF/ICSI treatments in combination with FET.

The incidence of live-birth defects was not higher, compared with the rate in those using the traditional ovarian stimulation regimen. The total rate of congenital malformation in live-born infants was 0.73% in our study, slightly lower than the 1.09% rate for abnormalities occurring in infants within 7 days after delivery, in People’s Republic of China, as reported in another study^51^. In fact, the study indicated that Utrogestan + hMG protocol is safe for live-born infants at birth. The reason why the rate of birth defects was lower in our study can be elucidated from the following aspects. First and foremost, women with maternal diseases or adverse environmental exposure during pregnancy was excluded from the study. In addition, all the participants underwent FET, which have been reported to have a lower risk of birth defects compared with fresh ET cycles^52^. Furthermore, induced abortion because of detectable malformations were excluded from the study. Fetal deformities tend to be timely diagnosed and terminated due to patients’ heightened awareness and concern in an IVF-conceived pregnancy.

A major limitation of our study is the retrospective design of this study and small sample size. Although no significant differences were found between the two groups in the rate of congenital anomalies, this may have been due to a type II error in view of the small sample size. Additionally, the data about the neonatal information were collected from parent questionnaires, rather than from direct access to medical records. However, the findings offer an insight into the safety of the use of Utrogestan for ovarian stimulation in combination with FET.

In conclusion, our preliminary study found that the data to date do not indicate an elevated rate of adverse neonatal outcomes after ovarian stimulation using Utrogestan + hMG protocol, but further study with larger populations is needed to confirm this finding. Application of Utrogestan for the prevention of premature LH surges has advantages of oral administration, user convenience, fee reduction and increased user compliance. These results will help the popularization and application of the new regimen as an oral alternative to GnRH analogue treatment, in combination with embryo cryopreservation.

## Materials and Methods

### Study Population and Design

A retrospective cohort study was conducted at the Department of Assisted Reproduction of the Ninth People’s Hospital of Shanghai Jiao Tong University School of Medicine (Shanghai, People’s Republic of China). The study was approved by the hospital’s ethics committee. Infertile couples, who underwent IVF or ICSI treatments with frozen-thawed embryo transfer (FET) using Utrogestan + hMG protocol, or the standard GnRH-a short protocol, were recruited at our center. Informed written consent was obtained from patients in accordance with the ethics committee protocol. The procedure about collecting neonatal outcome data was was extensively described elsewhere^12^. Briefly, a telephone interview with couples was the primary way, and family planning service agencies were contacted when connection with couples was lost.

These patients underwent the FET procedures from January 1, 2014 to December 31, 2014. Infants born to mothers with reported maternal diseases, such as gestational diabetes mellitus, hypertension, and thyroid disorders, or adverse environmental exposure during pregnancy, were excluded from this analysis because of the possible association of these factors with birth defects. The final data, involving 546 live-born infants, were stratified into groups according to the protocol of ovarian stimulation: 293 births after Utrogestan + hMG protocol and 253 births after the standard GnRH-a short protocol. The study design and participant selection procedure are presented in Fig. 1.

### Treatment

HMG 150 to 225 IU (Maanshan Pharmaceutical Trading Co., Anhui, China) and Utrogestan (Laboratories Besins International, Paris, France) 100 mg twice a day were administered from MC3 until the trigger day. In the study group After 6 to 7 days, follicular monitoring was performed using a transvaginal ultrasound examination to record the number of developing follicles,while serum FSH, LH, E_2_, andprogesterone concentrations were measured. When 3 dominant follicles reached 18mm in diameter, the final stage of oocyte maturation was triggered using triptorelin 0.1 mg (Decapeptyl, Ferring Pharmaceuticals, Germany). Transvaginal ultrasound-guided oocyte retrieval was conducted 34 to 36 hours after the trigger. All follicles with diameters of more than 10 mm were retrieved.

In the control group,triptorelin 0.1 mg daily beginning on MC_2_ and hMG 150 to 225 IU daily beginning on MC_3_ were administered. The ultrasound examination was started at MC_9–11_ and repeated every 2–4 days. The serum hormone level tests were performed on the same days as the ultrasound exams. The hMG dose was adjusted according to the follicle development and the serum hormone levels. When the dominant follicles reached 18mm in diameter, the final stage of oocyte maturation was induced with an intramuscular injection of hCG 3000 IU. Oocyte retrieval was conducted 34 to 36 hours later.

Fertilization of the aspirated oocytes was performed by either IVF or ICSI, depending on the semen parameters. According to the number and regularity of blastomeres and the degree of embryonic fragmentation, good-quality embryos (including grade 1 and grade 2, 8-cell embryos) were frozen by vitrification on the third day following the oocyte retrieval, and non-top-quality embryos were placed in extended culture, of which good morphological grade blastocysts were frozen on day 5 or day 6.

The method of embryo and endometrium synchronization during a natural cycle, an induced ovulation cycle, or an artificial cycle; and the timing of ET are extensively described elsewhere (11–13). Once pregnancy was achieved, the progesterone supplement was continued until 10 weeks of gestation.

### Pregnancy and neonatal outcomes

Pregnancy outcomes included clinical pregnancy rate, early miscarriage rate, late miscarriage rate, implantation rate, ongoing pregnancy rate, ectopic pregnancy rate and live birth rate. Clinical pregnancy was defined as the presence of a gestational sac with fetal heart activity during ultrasound examination 7 weeks after FET. The early miscarriage rate was defined as the proportion of patients with spontaneous pregnancy termination before the gestational age of 12 weeks. The late miscarriage rate was defined as the proportion of patients with pregnancy termination after the gestational age of 12 weeks. The multiple pregnancy rate was defined as the number of patients with two or more gestational sacs divided by the number of pregnant patients. The implantation rate was defined as the number of gestational sacs divided by the number of embryos transferred. The live-birth rate was defined as the proportion of patients with live birth among all transfer cycles.

Neonatal outcomes included preterm birth (PTB), low birth weight (LBW), gestational age, mode of delivery, early neonatal mortality and congenital malformations.

PTB was defined as deliveries before 37 weeks of gestation. LBW was defined as birth weights within 2500 g. Early neonatal death was defined as the death of a live-born baby within 7 days of birth. Congenital anomalies were defined as all structural, functional, and genetic anomalies diagnosed in aborted fetuses, at birth or in the neonatal period. Congenital malformations were classified using the International Classification of Diseases Q codes (Q00–Q99) as conditions registered in the International Statistical Classification of Diseases and Related Health Problems^12^.

### Statistical Analysis

Statistical analyses were carried out using SPSS 19.0 software (SPSS, Inc.). Data were presented as means ± SD; qualitative data were presented as percentages. Data were analyzed using the Student’s t-test, Mann-Whitney U-test, and, x^2^-test where appropriate. The Mann-Whitney U-test was used for the variables of non-normal distribution. P < 0.05 was considered statistically significant.
